# The complete mitochondrial genome of *Cirriformia* cf. *tentaculata* (Polychaeta, Cirratulidae) from Dok-Do, Korea

**DOI:** 10.1080/23802359.2017.1407694

**Published:** 2017-11-25

**Authors:** Hyun Ki Choi, Hyung June Kim, Donguk Han, Yong Rock An, Hana Kim

**Affiliations:** a Department of Taxonomy and Systematics, National Marine Biodiversity Institute of Korea, Seocheon-gun, Republic of Korea;; b Department of Biological Sciences, Inha University, Incheon, Republic of Korea

**Keywords:** Complete mitogenome; *Cirriformia tentaculata*, Cirratulidae, Polychaeta

## Abstract

Mitogenome sequence of *Cirriformia* cf. *tentaculata* (Terebellida, Cirratulidae), a species of marine polychaete worm, was determined in this study. This is the first mitogenome reported for genus *Cirriformia* and family Cirratulidae. This complete mitogenome is 15,495 bp in length, containing 13 protein coding genes (PCGs), two ribosomal RNAs (rRNAs), and 23 transfer RNAs (tRNAs). The mitogenome of *Cirriformia* cf. *tentaculata* has high A + T content of 59.5% (A, 29.5%; C, 26.9%; G, 13.6%; T, 30.0%). In neighbour-joining (NJ) analysis, *Cirriformia* cf. *tentaculata* is clustered with the components of order Terebellida.

*Cirriformia tentaculata* (Montagu, 1808), a species of marine polychaete worms belonging to family Cirratulidae Carus, 1863, has been considered as a typical cosmopolitan species (Çinar 2007). However, this opinion has been challenged and recently recognized as invalid. Some *Cirriformia* species*, C. chicoi* (Magalhães et al. 2014) and *C. capixabensis* (Magalhães et al. 2014) have been newly revealed through taxonomic re-examination of materials originally regarded as *C. tentaculata* (Magalhães et al. 2014). Although *Cirriformia tentaculata* is well-known in Korean polychaete fauna (Paik 1989), re-examination for materials assigned to *C. tentaculata* from this region is needed based on the study of Magalhães et al. (2014). The present study is a preliminary work for the verification of *Cirriformia* species from Korean waters based on molecular taxonomy, and we provide basic molecular information, a complete mitochondrial genome (mitogenome) of *Cirriformia* cf. *tentaculata* for the further study.

Samples were collected from subtidal habitats at Dok-Do in East Sea (Sea of Japan) by SCUBA diving. These voucher specimens were deposited in National Marine Biodiversity Institute of Korea (MABIK NA00146076-NA00146078). Genomic DNA was isolated from muscle tissue. Mitogenome sequences were obtained using Illumina Hiseq2000 sequencing platform (Macrogen, Seoul, Korea). These sequences were assembled and annotated in comparison with mitogenome sequences of species belonging to order Terebellida previously reported by Zhong et al. (2008) using Geneious 9.1.8 (Kearse et al. 2012). Additionally, we used the mitochondrial genome annotation (MITOS) server (Bernt et al. 2013) and tRNAscan-SE server (Lowe and Chan 2016) for annotation. Phylogenetic tree was constructed using MEGA6 (Tamura et al. 2013).

The complete mitogenome of *Cirriformia* cf. *tentaculata* (GenBank accession number MG267394) is 15,495 bp in length, containing 13 protein coding genes (PCGs), two ribosomal RNAs (rRNAs), and 23 transfer RNAs (tRNAs). The mitogenome of *Cirriformia* cf. *tentaculata* has high A + T content of 59.5% (A, 29.5%; C, 26.9%; G, 13.6%; T, 30.0%). All 13 PCGs use ATG as an initiation codon. Nine PCGs (*cox1*, *cox2, cox3, cytb, nad4l, nad4, nad1, nad3,* and *nad2*) have TAA as stop codon. Three PCGs (*atp8*, *atp6,* and *nad6*) use TAG as stop codon, while one PCG (*nad5*) has an incomplete stop codon, T. The lengths of tRNA genes are 60–68 bp and all tRNAs have the typical clover leaf structure except tRNA^met^ and tRNA^Ser^_UCN_. These two tRNAs have a reduced DHU arm.

Molecular phylogenetic position of *Cirriformia* cf. *tentaculata* was determined using concatenated sequences of 13 PCGs by neighbour-joining (NJ) analysis based on K2P model in MEGA6. Although *Cirriformia* cf. *tentaculata* is clustered with components of order Terebellida (the parent group of family Cirratulidae) in NJ tree (Figure 1), detailed phylogenetic relationship for this species needs additional analysis due to the lack of mitochondrial sequences from its congeneric species.

**Figure 1.**
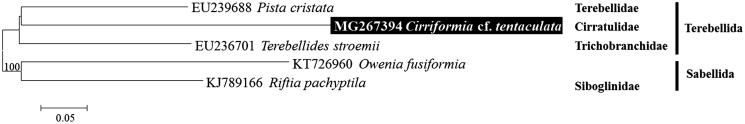
Neighbour-joining (NJ) tree based on the mitogenome sequences of *Cirriformia* cf. *tentaculata* with two other related species in Terebellida. *Owenia fusiformis* and *Riftia pachyptila* derived from Sabellida were used as outgroup for tree rooting. Numbers above the branches indicate NJ bootstrap values from 1000 replications.
